# Effects of Online and Face-to-Face Intuitive Eating Interventions on Body Image and Eating Behaviors among Women in China: A Feasibility Study

**DOI:** 10.3390/nu14091761

**Published:** 2022-04-22

**Authors:** Ziyue Cheng, Xueyan Gao, Chengyang Yang, Anna Brytek-Matera, Jinbo He

**Affiliations:** 1 School of Humanities and Social Science, The Chinese University of Hong Kong, Shenzhen 518172, China; ziyuecheng@link.cuhk.edu.cn (Z.C.); xueyangao@link.cuhk.edu.cn (X.G.); chengyangyang@link.cuhk.edu.cn (C.Y.); 2 Institute of Psychology, University of Wroclaw, 50-527 Wroclaw, Poland; anna.brytek-matera@uwr.edu.pl

**Keywords:** intuitive eating, disordered, body image, feasibility study, intervention, Chinese women

## Abstract

Body dissatisfaction and eating disorders have become major global concerns, including in Asian populations. Few studies have examined intervention effects on body dissatisfaction and disordered eating in China, especially for interventions with positive psychological perspectives (e.g., intuitive eating). In this pilot study, 66 women participated in an eight-module intuitive eating intervention delivered online (*n* = 42; mean age, 30.74 years) and face-to-face (*n* = 24; mean age, 19.46 years) for 8 weeks. Measures of body image and eating behaviors were used to assess the intervention’s feasibility, acceptability, and initial efficacy. Linear mixed models were used to analyze the data. The intervention had significant effects on both groups, promoting positive body image and intuitive eating and reducing negative body image and disordered eating behaviors. The effects of the online and face-to-face interventions did not differ significantly. Thus, whether delivered online or face-to-face, an intuitive eating intervention may effectively improve Chinese women’s body image and eating behaviors. However, the efficacy of the intuitive intervention in the Chinese context should be confirmed in future studies with designs in randomized control trials.

## 1. Introduction

Body dissatisfaction and eating disorders have become contemporary global mental health issues [1,2], including in China [3,4]. A survey showed that most female Chinese college students are regarded as underweight (based on body mass index (BMI) range) as ideal and tended to adopt strict dieting rules to lose weight, thereby increasing the risks of body dissatisfaction and eating disorders [5]. However, although dieting may lead to moderate weight loss in the short term, it may promote weight gain in the long term [6]. It may also increase psychological distress or clinical impairment, preoccupation about food or weight, depression, weight fluctuation, and low self-esteem [7,8,9,10]. Thus, to promote healthy eating and a positive body image, intuitive eating intervention was developed [11].

Intuitive eating is described as a dynamic process of the unity of body, food, and mind [12]. It is an adaptative way for individuals to regulate food intake based primarily on internal cues, such as hunger and satiety. Thus, it is based on close associations with interoceptive signals and inner sensory consciousness. The intuitive eating intervention, developed by Tribole and Resch in 1995, has 10 guiding principles (e.g., rejecting the diet mentality, honoring your hunger, and feeling your fullness) [6]. Intuitive eating can be practiced in multiple ways. People can grasp its concept and practice healthy eating methods by reading books, undergoing therapeutic counseling, or taking training courses to overcome dieting and eating problems and regain a healthy eating style [12].

Intuitive eating has been proven to improve health outcomes, such as cholesterol levels, blood pressure, and insulin sensitivity [13,14,15]. In addition, physical acceptance- and intuition-based interventions have been shown to effectively reduce the risk of eating disorder development, the internalization of thoughts about extreme weight loss, dietary constraints, and psychological disorders [16]. Intuitive eating has been correlated with positive outcomes such as BMI reduction [17,18]. A 10-week intuitive eating and mindfulness training intervention implemented with 93 college students and university staff members in the United States increased female participants’ body appreciation and intuitive eating and reduced the odds of problematic eating behavior [19].

A focus on cultural factors related to body image and eating disorders is also relevant. Different cultural backgrounds are associated with various dieting behaviors (e.g., dairy restraint) [20]. In a cross-cultural study, Chinese women were found to have fewer eating disorders than non-Hispanic white women, suggesting that Asian dietary culture might be considered a protective factor [21]. However, Chinese women had significantly less body image satisfaction than non-Hispanic white women, implying the existence of further cultural differences associated with body image [21]. Moreover, clinical therapy for eating disorders may differ among cultural settings. Interventions focusing on young women’s body image and disordered eating are still in the early stages in China [22]. A recent review demonstrated that three main clinical treatments (a family-based intervention model, cognitive behavioral therapy, and dialectical behavior therapy) efficiently addressed Chinese patients’ eating disorders, but that these treatments are lengthy and costly for Chinese patients to complete [22]. Thus, low-cost, efficient, and effective intervention courses for Chinese women are needed.

To our knowledge, no study has evaluated intuitive eating interventions implemented in China. Such interventions need to be validated in the Chinese context before being placed in widespread use. Moreover, the strengths of digital mental health interventions have been demonstrated [23]. With the 2019 coronavirus disease (COVID-19) pandemic, the merit of examining whether online interventions could be recommended more globally has become apparent. Thus, we conducted this pilot study with two main aims: (1) to investigate an intuitive eating intervention’s preliminary efficacy on Chinese women’ eating behaviors and body image; and (2) to compare the effects of online and face-to-face implementation of the intervention. The findings of this study will aid the introduction of a new healthy eating behavior intervention in China. They can be used to develop further formal experiments and digital health applications.

## 2. Materials and Methods

### 2.1. Participants and Procedure

Ethical approval for this study was obtained from the Research Ethics Committee of the Chinese University of Hongkong, Shenzhen (No. EF20210717001). Participants were recruited via online and offline posters. The offline poster was displayed on campus for 2 weeks in The Chinese University of Hong Kong, Shenzhen, China. The online poster was posted on Chinese social media (e.g., Weibo) for 2 weeks. Individuals interested in participating in the study scanned the QR code on the poster to fill in the survey and provide demographic and contact information. Our research team sent an invitation email to all individuals who took the survey. Participants were recruited who met the inclusion criteria: (1) Chinese nationality, (2) adult women aged ≥ 18 years, (3) no serious physical or mental disease (including a diagnosed eating disorder). In addition, as both women with and without disordered eating can benefit from intuitive eating interventions [24,25], we did not set an inclusion criterion on disordered eating symptoms. A total of 139 women registered to participate; however, after excluding those not meeting the inclusion criteria, 87 remained. The included participants were assigned to online and face-to-face intervention groups based on their convenience. Specifically, those who lived or studied in person at the university (36 participants) would attend the face-to-face intervention sessions, while the remaining (51 participants) would attend the online intervention sessions. Online or written informed consent was obtained from all participants.

### 2.2. Research Design

As this study was designed as a pilot test of the primary effects of intuitive eating intervention on eating behaviors and body image, similarly to previous pilot studies [24,25], we used a single arm pretest/posttest design (i.e., without a control group). The research team was composed of one mentor and two assistants. All team members completed a training program and prepared a course manual, slide presentations, and other materials for the intervention. Two assistants measured the height and weight of participants in the face-to-face intervention group using a standardized balance scale and tapeline before and after intervention implementation. Participants in the online intervention group self-measured and self-reported their height and weight at the same timepoints. The intervention was provided to all subjects in eight weekly modules. Each face-to-face session lasted about 40 min and was delivered by the research team. The modules were delivered via the Ding Talk application as 20-min videos, all recorded by the same researcher, to participants in the online intervention group. All participants underwent baseline assessment and post-test anonymously before and after participating in the formal intervention.

### 2.3. Intervention Characteristics

The intuitive eating interventions were implemented from September to December 2021 in Shenzhen, China. The intervention modules were based on the intuitive eating workbook written by Evelyn and Tribole [26] and guiding videos from the official website of (www.intuitiveeating.org accessed on 1 June 2021). The process of intuitive eating intervention preparation modules were separately demonstrated into several parts: (1) collected and concluded about the models or theories about intuitive eating; (2) collection and analysis of studies, theories, and models focusing on body image and eating behaviors among women in China; (3) invitation of professional experts to provide course-designing advice and act as counselors during the intervention; (4) intervention course design; (5) preparation of the intervention manual, teaching plan, and slide presentations; (6) recording and editing of the videos for the online modules; (7) recruitment of three volunteers for a trial of the intervention and collection of their feedback about the modules; and (8) modification and confirmation of the final modules. The eight modules are summarized in Table 1.

### 2.4. Measures

#### 2.4.1. Short-Form Eating Disorder Examination Questionnaire

We used the short form of the Eating Disorder Examination Questionnaire (EDE-QS) [27], which has been translated and validated the Chinese population (i.e., C-EDE-QS) [28]. C-EDE-QS has 12 items which were rated on a 4-point scale ranging from 0 to 3. The total score of the C-EDE-QS represents the severity of disordered eating symptomatology. In the present study, the internal consistency coefficient for this instrument in the baseline survey was 0.87.

#### 2.4.2. Intuitive Eating Scale

The Intuitive Eating Scale-2 consists of items in four dimensions: unconditional permission to eat, eating for physical rather than emotional reasons, reliance on internal hunger and satiety cues to determine when and how much to eat, and body–food choice congruence [29]. Intuitive eating was incorporated into one dimension of assessing eating behavior. In the present study, we used the Chinese version of the Intuitive Eating Scale-2 (C-IES-2), which has been validated in a Chinese population [30]. The C-IES-2 consists of 23 items for which responses are provided on a 5-point scale ranging from 1 (totally disagree) to 5 (totally agree). In the present study, Cronbach’s alpha coefficient for this instrument in the baseline survey was 0.83.

#### 2.4.3. Body Image Acceptance and Action Questionnaire

The Body Image Acceptance and Action Questionnaire (BI-AAQ) is used to evaluate body image flexibility. Body image flexibility was incorporated into one dimension of measuring body image. It consists of 12 items for which responses are provided on a seven-point Likert scale ranging from 1 (never true) to 7 (always true) [31]. The Chinese version of the BI-AAQ, which has sound psychometric properties [32], was used in this study. Cronbach’s alpha coefficient for this instrument in the baseline survey was 0.90.

#### 2.4.4. Inflexible Eating Questionnaire

The Inflexible Eating Questionnaire (IEQ) consists of 11 items for which responses are provided on a 5-point Likert scale ranging from 1 (totally disagree) to 5 (totally agree). Higher scores reflect more severe inflexible eating rules. Eating inflexibility was included into one dimension of evaluating subjects’ eating behavior. The Chinese version of the IEQ, which has shown good performance with Chinese participants [33], was used in this study. Cronbach’s alpha coefficient for this instrument in the baseline survey was 0.89.

#### 2.4.5. Functionality Appreciation Scale

The Functionality Appreciation Scale (FAS) was designed to demonstrate the conceptualization of functionality appreciation. It has 7 items for which responses are provided on a 5-point Likert scale ranging from 1 (totally disagree) to 5 (totally agree) [34]. Functionality appreciation was incorporated into one dimension of assessing body image. The Chinese version of the FAS [35] was used in the current study. Cronbach’s alpha coefficient for this instrument in the baseline survey was 0.92 in current study.

#### 2.4.6. Clinical Impairment Assessment 3.0

The Clinical Impairment Assessment 3.0 (CIA 3.0) is a self-reported measure of the severity of psychosocial impairment duo to eating disorder symptoms [36]. It consists of 16 items for which responses are provided on a 4-point Likert scale ranging from 0 (not at all) to 3 (a lot). Clinical impairment was incorporated into one dimension of measuring subjects’ psychosocial impairment. In the present study, we used the Mandarin Chinese translation of the CIA 3.0 [37]. Internal consistency coefficient in the baseline survey was 0.96.

#### 2.4.7. Body Dissatisfaction Subscale of the Eating Disorder Inventory

The Eating Disorder Inventory (EDI), developed in 1983 by Garner et al. [38], is used to identify individuals’ tendencies toward anorexia nervosa or bulimia nervosa based on a typical psychological symptom cluster. Item responses are structured by a 6-point Likert-type scale ranging from 1 (never) to 6 (always). We used the Mandarin Chinese version of the 9-item body dissatisfaction subscale of the EDI [39] in this study. Body dissatisfaction was included into one dimension to evaluate body image in this study. Cronbach’s alpha coefficient for this instrument in the baseline survey was 0.91.

#### 2.4.8. Body Appreciation Scale-2

The Body Appreciation Scale-2 (BAS-2) contains of 10 items for which responses are provided on a 5-point Likert scale ranging from 1 (never) to 5 (always) [40]. Body appreciation was incorporated into one dimension of measuring the body image of the participants. The Chinese version of the BAS-2, which has shown good psychometric properties [41], was used in the current study. Cronbach’s alpha coefficient in the baseline survey was 0.93.

### 2.5. Data Analysis

All data analyses were carried out with R (version 4.1.3). We used linear mixed models (LMMs) implemented with the lmer Test package [42] to analyze the effects of the online and face-to-face interventions separately, similarly as in a previous pilot study [25]. Cohen’s *d* was used as the effect size estimate, and was calculated using the lme.dscore function with the EMA tools package [43]. The covariates were age and BMI. Cohen’s *d* values < 0.2 were considered to represent small effects, those close to 0.5 were considered to represent medium effects, and those >0.8 were considered to represent large effects.

## 3. Results

### 3.1. Sample Characteristics

**Figure 1:**
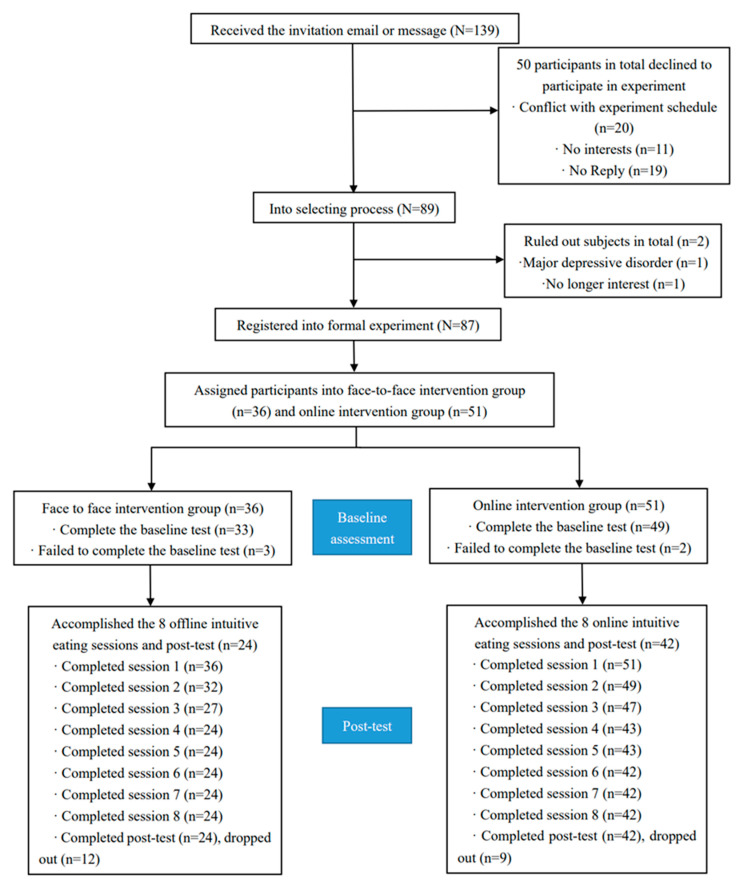
Flow of Study Participation.

Of the 87 included participants, 36 participants attended the face-to-face intervention group, and 51 attended the online intervention group. Sixty-six participants (online, *n* = 42; face-to-face, *n* = 24) completed the baseline assessment, intervention, and post-test (Figure 1). The main reasons for dropout were schedule conflicts and loss of interest. The demographic characteristics of the final sample are summarized in Table 2. Specifically, the mean age of participants in the online intervention group was 30.74 (standard deviation (SD), 8.25) years, and the mean age of face-to-face intervention participants was 19.46 (SD, 1.29) years.

### 3.2. Intervention Effects

#### 3.2.1. Eating Behaviors

The LMM results are provided in Table 3. Relative to baseline, the average eating dis-order symptom levels as measured by the EDE-QS had decreased significantly in the online (*F* = 20.80, *p* < 0.001, Cohen’s *d* = 1.42) and face-to-face (*F* = 6.68, *p* < 0.05, Cohen’s *d* = 1.09) intervention groups after the intervention. In both groups, the mean intuitive eating levels as measured by the IES-2 had increased significantly (online: *F* = 29.52, *p* < 0.001, Cohen’s *d* = 1.69; face-to-face: *F* = 30.69, *p* < 0.001, Cohen’s *d* = 2.28) and the mean eating inflexibility levels as measured by the IEQ had decreased significantly (online: *F* = 18.11, *p* < 0.001, Cohen’s *d* = 1.32; face-to-face: *F* = 23.49, *p* < 0.001, Cohen’s *d* = 1.98).

#### 3.2.2. Body Image

After the intervention, body image inflexibility as measured by the BI-AAQ had decreased significantly among online intervention participants (*F* = 11.06, *p* < 0.01, Cohen’s *d* = 1.03), but not in the face-to-face intervention group (*F* = 1.62, *p* > 0.05, Cohen’s *d* = 0.52). The same pattern was observed for functionality appreciation as measured by the FAS (online: *F* = 9.31, *p* < 0.01, Cohen’s *d* = 0.94; face-to-face: *F* = 3.60, *p* > 0.05, Cohen’s *d* = 0.78). Body dissatisfaction as measured by the EDI-BD had decreased significantly in the online (*F* = 30.37, *p* < 0.001, Cohen’s *d* = 1.70) and face-to-face (*F* = 17.92, *p* < 0.001, Cohen’s *d* = 1.73) intervention groups. Body appreciation as measured by the BAS-2 had increased in both groups (online: *F* = 36.64, *p* < 0.001, Cohen’s *d* = 1.85; face-to-face: *F* = 19.59, *p* < 0.001, Cohen’s *d* = 1.83).

#### 3.2.3. Psychosocial Impairment

The degree of clinical impairment as measured by the CIA-3.0 decreased significantly between baseline and post-intervention in the online (*F* = 12.88, *p* < 0.001, Cohen’s *d* = 1.11) and face-to-face (*F* = 5.81, *p* < 0.05, Cohen’s *d* = 0.98) intervention groups.

### 3.3. Interaction between Intervention Effects and Intervention Type

No significant interaction effects were observed between any intervention conditions (Table 4). This result suggests that the effects of the online and face-to-face interventions did not differ significantly.

## 4. Discussion

The intuitive eating intervention implemented in this study had positive effects on Chinese women’s eating behaviors and body image, demonstrating the preliminary efficacy of the intuitive eating intervention in the Chinese context. In addition, the outcomes were similar for the online and face-to-face groups.

Participants’ eating behaviors were assessed in terms of eating disorder symptoms, intuitive eating, and eating inflexibility. Concerning eating behaviors, the results suggest that discussion of the concept of eating with unconditional permission in the intervention reduced individuals’ dieting and other eating problems [44]. In the intuitive intervention, the negative effects of dieting (e.g., weight rebound, mood swings) were emphasized and discussed repeatedly, which might lead to a change of participants’ cognition about dieting and would eventually help participants abandon dieting and then reduce the negative effects due to dieting [12]. In addition, the refusal to engage in emotional eating behavior was highly recommended in the intervention, and has been found to directly reduce the frequency of gluttonizing or binge eating due to emotional problems [45]. The intervention content on attention to hunger and satiety signals helped participants to feel their body’s changes and to adjust their eating behaviors based on signals from their body, which could reduce the probability of binge eating and aid the maintenance of healthy eating habits [12]. A lack of interoceptive self-awareness is common among individuals with eating disorders [46]. Overall, participants’ intuitive eating had improved distinctly after the intervention, reflecting their practice and application of healthy eating habits and methods in real life.

In addition, the intervention reduced participants’ body dissatisfaction and increased their functional appreciation. These results confirm the finding that the control of unhealthy eating behaviors and cognition about one’s appearance or weight (emphasized in the intervention) reduces the risk of eating disorder symptoms [19]. Moreover, such improvements increase individuals’ ability to achieve healthier lifestyles (e.g., with more exercise and balanced nutritional intake) and proper cognition about their own bodies (i.e., trusting and caring) [24]. Only the online intervention significantly reduced body image inflexibility in this study; this outcome may have been influenced by the younger age of the face-to-face intervention group, which might be associated with stricter rules for and expectations about one’s appearance [47]. However, this assumption needs to be tested in a future study. Overall, the intuitive eating intervention had a positive effect on female Chinese participants’ body image.

The intervention reduced participants’ clinical impairments related to eating disorder symptomatology. Intuitive eating has been shown to reduce dieting and overfocus on weight/food behaviors, and thus at least partially address the clinical impairments related to eating disorder symptomatology.

We found no significant difference between the online and face-to-face interventions after controlling for covariates. The equivalent effectiveness of such interventions is in line with previous findings [48]. The online delivery of an eating and body image intervention appears to be promising in the Chinese context, especially in settings such as the COVID-19 pandemic.

The current study has several limitations. First, because it was a pilot feasibility study, it did not include a control group; thus, the intervention’s effects should be confirmed further with randomized control trials. Second, the attrition rate in the face-to-face intervention group was high (12 of 36 participants), as this intervention was conducted mainly with undergraduate students on school days, raising conflicts due to exams and other study-related activities. This high attrition rate may have reduced the power to detect certain effects in the current study (e.g., the effect on body image inflexibility was medium, but not significant). Finally, all participants in this study were female; eating and weight disorders among Chinese males are also public health concerns and warrant more research [49,50]. Thus, future studies should explore the feasibility of the intuitive eating intervention for Chinese males and identify any significant gender differences. In addition, we used different ways to assess participants’ BMI, with the BMI for the face-to-face group and online group based on objectively measured and self-reported weight and height, respectively. Since BMI based on self-reported weight and height may be distinct from BMI based on objectively measured weight and height, the findings regarding the online intervention might be affected. However, considering BMI was not a focus in the current study and was merely used as a covariate in the data analysis, the issue of assessment of BMI for the online group might have limited influence on the findings of the current study.

The significant effects of the intuitive eating intervention among Chinese women in this study inspires us to conduct further research. For example, the intervention may support Chinese women with eating disorders or body image issues to regain healthy lifestyles. In addition, given the complexity and instability of individual factors, more consideration of indicators such as participants’ interpersonal relationships would be of value. As the online intervention results confirmed the feasibility and effectiveness of remote interventions of this type, which could serve as a more-flexible alternative to face-to-face therapy in settings such as the COVID-19 pandemic, we would also like to explore online intervention module delivery further (e.g., assessing the effects of live broadcasts in addition to videos).

## 5. Conclusions

This pilot study demonstrated the preliminary efficacy of the intuitive eating intervention on Chinese women’s eating behaviors and body image. The online and face-to-face interventions were similarly effective. However, considering that this is a pilot study without a control condition, individuals should be cautious about interpreting the preliminary efficacy. However, the positive results of this pilot study support developing intuitive interventions in the Chinese context with randomized control trials.

## Figures and Tables

**Table 1:** Intuitive eating intervention modules and overview of their content.

Session	Module	Content
1	Meet intuitive eating	Basic introduction to the concept of intuitive eating, meeting course participants
2	Reject dieting	Principle 1: reject the diet mentality. Introduction to dieting’s harm and costs, explanation of the dieting mental cycle
3	Accept your hunger	Principle 2: honor your hunger. Instruction about hunger and internal body sign awareness
4	Let’s make peace with food	Principle 3: make peace with food. Overview of how to make peace with food
5	Deal with the food police	Principle 4: challenge the food police. Explanation of the food police concept and instruction about how to deal with it
6	Learn to feel fullness and satisfaction	Principles 5 and 6: feel your fullness and discover the satisfaction factor. Instruction about the internal and external appearance of fullness, exploration of self-satisfaction factors
7	Learn to respect your body with the help of making peace with food	Principles 7 and 8: cope with your feelings without using food and respect your body. Exploration of the harm of emotional eating behaviors, self-care, trust, and respect for your body
8	Practice and application	Principles 9 and 10: Exercise: feel the difference and honor your health; gentle nutrition. Exploration of pleasurable exercise activities and the advantages and disadvantages of exercise. Instruction on nutrition for food selection. Future plan development

**Table 2:** Sample characteristics.

Characteristics	Face-to-Face Group		Online Group	
	M (SD)	N (%)	M (SD)	N (%)
Age(years)	19.46(1.29)		30.74(8.25)	
Origin Area				
Urban		23 (95.8%)		39(92.9%)
Rural		1(4.2%)		3(7.1%)
Ethnicity				
Main		22(91.7%)		39(92.9%)
Minority		2(8.3%)		3(7.1%)
Baseline BMI	23.05(4.66)		23.44(3.72)	
Post-test BMI	23.03(4.63)		23.04(3.75)	

Note. M, mean; SD, standard deviation; BMI, body mass index (weight (kg)/height (m)^2^).

**Table 3:** Intervention effects.

Outcomes	Face-to-Face Group				Online Group			
	Before M(SD)	After M(SD)	*F* Test	Cohen’s *d*	Before M(SD)	After M(SD)	*F* Test	Cohen’s *d*
Eating disorder symptoms	12.79(8.59)	8.46(8.40)	*F*(1, 22.46) = 6.68 *	1.09	12.78(5.78)	8.48(6.73)	*F*(1, 41.48) = 20.80 ***	1.42
Intuitive eating	72.71(13.30)	87.46(16.40)	*F*(1, 23.69) = 30.69 ***	2.28	74.29(10.53)	84.93(9.94)	*F*(1, 41.33) = 29.52 ***	1.69
Body image inflexibility	3.38(1.66)	3.08(1.85)	*F*(1, 24.23) = 1.62	0.52	4.04(1.25)	3.36(1.33)	*F*(1, 41.64) = 11.06 **	1.03
Eating inflexibility	3.08(0.81)	2.29(0.96)	*F*(1, 23.92) = 23.49 ***	1.98	3.18(0.70)	2.70(0.68)	*F*(1, 41.41) = 18.11 ***	1.32
Functionality appreciation	4.39(0.62)	4.64(0.53)	*F*(1, 23.55) = 3.60	0.78	4.21(0.64)	4.49(0.47)	*F*(1, 41.67) = 9.31 **	0.94
Clinical impairment	13.88(12.87)	9.96(12.23)	*F*(1, 24.16) = 5.81 *	0.98	13.69(9.87)	8.48(8.99)	F(1, 41.57) = 12.88 ***	1.11
Body dissatisfaction	38.17(10.96)	30.83(13.34)	*F*(1, 23.84) = 17.92 ***	1.73	41.78(8.98)	35.90(11.14)	*F*(1, 42.08) = 30.37 ***	1.70
Body appreciation	3.23(1.04)	3.97(0.92)	*F*(1, 23.30) = 19.59 ***	1.83	3.10(0.82)	3.60(0.85)	*F*(1, 42.99) = 36.64 ***	1.85

Note. * *p* < 0.05, ** *p* < 0.01, *** *p* < 0.001. M, mean; SD, standard deviation.

**Table 4:** Interaction between intervention effects and type (online vs. face-to-face).

Variables	*F* Test	Cohen’s *d*
Eating disorder symptoms	*F*(1, 63.05) = 0.01	0.02
Intuitive eating	*F*(1, 64.07) = 1.96	0.35
Body image inflexibility	*F*(1, 64.08) = 0.27	0.28
Eating inflexibility	*F*(1, 64.00) = 2.88	0.42
Functionality appreciation	*F*(1, 64.15) = 0.15	0.04
Clinical impairment	*F*(1, 63.98) = 0.20	0.11
Body dissatisfaction	*F*(1, 64.15) = 0.89	0.24
Body appreciation	*F*(1, 64.28) = 2.62	0.40

## Data Availability

The data in the present study are available from the corresponding author on reasonable request.
